# An evolutionary explanation for antibiotics’ association with increased colon cancer risk

**DOI:** 10.1093/emph/eoac018

**Published:** 2022-04-29

**Authors:** Konstantinos Voskarides

**Affiliations:** Department of Basic and Clinical Sciences, University of Nicosia Medical School, Nicosia, Cyprus

**Keywords:** natural selection, MSH2, MLH1, DNA repair, cancer evolution, adaptation

## Abstract

More than 10 studies have confirmed the association of antibiotic overuse with colorectal cancer. The exact cause is unknown, but most authors hypothesize that disturbance of colon microbiota is the main culprit. In this commentary, an evolutionary explanation is proposed. It is well known that antibiotics can induce antibiotic resistance in bacteria through selection of mutators—DNA mismatch repair deficient (dMMR) strains. Mutators have an increased survival potential due to their high mutagenesis rate. Antibiotics can also cause stress in human cells. Selection of dMMR colon cells may be advantageous under this stress, mimicking selection of bacterial mutators. Concomitantly, mismatch repair deficiency is a common cause of cancer, this may explain the increased cancer risk after multiple cycles of oral antibiotics. This proposed rationale is described in detail, along with supporting evidence from the peer-reviewed literature and suggestions for testing hypothesis validity. Treatment schemes could be re-evaluated, considering toxicity and somatic selection mechanisms.

**Lay Summary:**

The association of antibiotics with colon cancer is well established but of unknown cause. Under an evolutionary framework, antibiotics may select for stress-resistant cancerous cells that lack mechanisms for DNA mismatch repair (MMR). This mimics the selection of antibiotic resistant ‘mutators’—MMR-deficient micro-organisms—highly adaptive due to their increased mutagenesis rate.

## ASSOCIATION OF ANTIBIOTICS WITH COLON CANCER

Multiple studies have found a positive association between overuse of antibiotics and risk for colorectal cancer, though the exact cause remains unknown. In this article, a connection with DNA mismatch repair (MMR) genes is speculated, under the view of somatic selection. Below, a detailed description of the most significant studies that found the antibiotics–cancer association is included. Studies are summarized in Table 1.

**Table 1.** Studies showing the association of antibiotics with colon or colorectal cancer

Study	Type of study	Type of cancer	Antibiotics	Odds ratio (CI)	Number of cases
[1]	Nation-wide cohort study	Colon	Any	1.15 (1.04–1.26)	7513
[2]	Case–control	Colon	Anti-anaerobic	2.31 (2.12–2.52)	3593
[3]	Case–control	Colorectal	Penicillins	1.04 (1.01–1.08) (per treatment)	20 990
[4]	Case–control	Colon	Ampicillin/amoxicillin	1.09 (1.05–1.13)	28 980
[5]	Case–control	Colorectal	Any	1.90 (1.61–2.19)	35 214
[6]	Meta-analysis	Colorectal	Broad-spectrum	1.70 (1.26–2.30)	73 550
[8]	Meta-analysis	Colorectal	Any	1.09 (1.02–1.17)	4 853 289 (all participants)
[9]	Meta-analysis	Colorectal	Any	1.20 (1.10–1.32)	3 408 312
[10]	Case–control	Colon	Any	1.17 (1.05–1.31)	40 545
[11]	Meta-analysis	Colorectal	Any	1.10 (1.01–1.18)	73 405
[49]	Case–control	Colon	Any	1.49 (1.07–2.07)	7903

CI, confidence intervals.

Kilkkinen *et al*. [1], using a Finnish registry of 3 112 624 individuals, aged 30–79 years, found a positive association of antibiotics use with several cancer organ sites, including prostate, breast, lung and colon. Wang *et al*. [2] surveyed 3593 colon cancer cases, 1979 rectal cancer cases and 22 288 controls, finding that the use of any anti-anaerobic antibiotic was associated with a higher risk of colon cancer (OR = 2.31, 95% CI: 2.12–2.52) and rectal cancer (OR = 1.69, 95% CI: 1.50–1.90) but without any obvious dose-dependent relationship. Boursi *et al*. [3] studied a total of 20 990 cases and 82 054 controls. They found an increased colorectal cancer risk with the use of penicillins, an increase by OR of 1.04 (95% CI: 1.01–1.08) per one additional treatment per year. No association was found with exposure to anti-viral or anti-fungal therapy. Zhang *et al*. [4] found an increased risk of colon cancer (UK cases) with antibiotics use in a dose-dependent fashion (28 980 colorectal cancer cases and 137 077 controls, *P* < 0.001). Along the same lines, Armstrong *et al*. [5] found a dose-dependent association with colorectal cancer for patients prescribed antibiotics in up to a 15-year timeframe (OR = 1.90, 95% CI: 1.61–2.19, *P* < 0.001). Simin *et al*. [6] in a huge meta-analysis (4.1 million individuals) found increased pooled colorectal cancer risk for individuals with any antibiotics exposure (OR = 1.17, 95% CI: 1.05–1.30), with particularly higher risk for broad-spectrum antibiotics (OR = 1.70, 95% CI: 1.26–2.30). Wan *et al*. [7] in a meta-analysis with 412 450 individuals in total found, after stratifying by type of antibiotic, that participants with extensive use of penicillin and anti-anaerobic antibiotics had 18% and 49% increased risk of colorectal cancer, respectively. In another meta-analysis by Qu *et al*., [8], more than 60 days of antibiotics use and five prescriptions of antibiotics were significantly associated with an elevated risk of colorectal cancer (OR = 1.09, 95% CI: 1.02–1.17). Sanyaolu *et al*. [9] searched MEDLINE, EMBASE and CINAHL databases for published observational studies. The final analysis included a total of 3 408 312 patients and found a weak association between antibiotic exposure and colorectal cancer when exposure was assessed cumulatively by the number of prescriptions (OR = 1.204, 95% CI: 1.097–1.322, *P* < 0.001) or duration of antibiotic exposure (OR = 1.168, 95% CI: 1.087–1.256, *P* < 0.001). The most recent studies were performed by Lu *et al*. [10] and Aneke-Nash *et al*. [11]. In the Swedish study by Lu *et al*. [10], 40 545 colorectal cancer cases and 202 720 controls were included. A positive association between frequent antibiotics use and colorectal cancer was found, especially for the proximal colon (adjusted OR for very high use vs no use = 1.17, 95% CI: 1.05–1.31). The study by Aneke-Nash *et al*. [11] was a meta-analysis of six papers. Individuals with high antibiotic exposure had a 10% higher risk of colorectal neoplasia than those with the lowest exposure (OR = 1.10, 95% CI: 1.01–1.18).

The association of antibiotics use and cancer is not restricted to colorectal tumors. Indicatively, two studies are referred here. The study of Kilkkinen *et al*. [1] found an association of antibiotics use with several forms of cancer. Boursi *et al*. [12] reported similar findings with antibiotic use being associated with several cancer types, studying 125 441 cases and 490 510 matched controls.

According to this vast amount of published data, there can be little doubt that antibiotics overuse is associated with increased colorectal cancer risk, often in a dose- or time-dependent manner. Antibiotic type was usually not found to be a significant parameter of this association; however, some studies agree on penicillin and anti-anaerobic antibiotics. Association of antibiotics with other cancer types probably needs further investigation. Is colorectal cancer–antibiotics a causal relationship? The obvious explanation is the disturbance of colon microbiota. This is the explanation that most authors give for their results. In this perspective article, an alternative evolutionary explanation will be discussed, related with the selection of DNA MMR-deficient cells under antibiotic stress. I would like to state here that I do not neglect the probable significance of microbiota to cancer. DNA MMR deficiency is probably a part of a complicate equation that drives to cancer.

## DNA MMR AND CANCER

**Figure 1.**
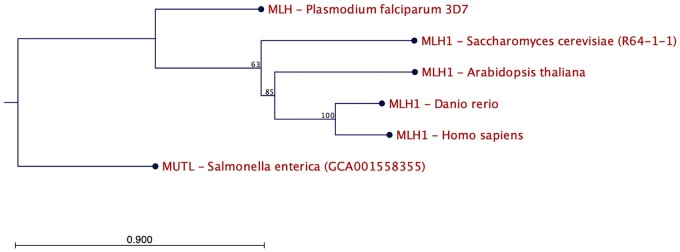
*MLH1* gene tree. *MLH1* orthologues exist in all five life kingdoms. Protein sequences were derived from Ensembl. Maximum-likelihood phylogeny method was used for the gene tree construction (CLC Main Workbench 21).

DNA MMR is considered one of the most important mechanisms of DNA damage repair and one of the most conserved molecular mechanisms in all living organisms (Fig. 1). MMR protein dimers recognize a variety of base–base and insertion–deletion mismatches [13], while other auxiliary proteins remove the wrong bases and DNA polymerase synthesizes the correct DNA sequence [13].

In humans, seven DNA MMR genes/proteins (*MLH1*,* MLH3*,* MSH2*,* MSH3*,* MSH6*,* PMS1* and *PMS2*) have been identified. For some of them, the exact function is not clear. Deficiencies in the MMR pathway are a frequent cause of carcinogenesis.

Most cancer cases are associated with somatic mutations in oncogenes and tumor suppressor genes. MMR genes are considered as tumor suppressor genes. Inherited neoplasias represent ∼ 5–10% of all cancer cases and usually follow an autosomal dominant model of inheritance. Mutations in the MMR genes are responsible for hereditary nonpolyposis colorectal cancer/Lynch syndrome (HNPCC/LS), and other cancer-predisposing Lynch variant syndromes. The majority of mutations in HNPCC/LS occur in *MSH2* and *MLH1* genes; however, mutations in other MMR genes are also implicated, such as *MSH6* and *PMS2* [14]. Additionally, somatic mutations in MMR genes are found in up to 15% of sporadic colorectal, gastric or endometrial carcinomas [15]. Specifically for colorectal cancers, 15% of tumors are deficient in DNA MMR, commonly due to loss of *MLH1* (9.8%) [16, 17]. Frequently, the defect found in *MLH1*-associated tumors is not a gene mutation but hypermethylation of the promoter. Promoter hypermethylation of *MLH1* is found in at least nine more cancer sites including gastric cancer (21.6%) [18] and oral squamous cell carcinoma (76%) [19].

Microsatellite instability (MSI) is considered the classical method for detecting MMR pathway deficiency in colorectal or other tumors. Microsatellites are short tandem repeats (STRs) that are found throughout the genome. The most common ones in the human genome are the dinucleotide repeats, especially (AC)n. In case of a deficient MMR pathway, genetic instability is detected as presence of multiple alleles (instead of two) per each analyzed STR in tumors’ DNA [20]. The National Cancer Institute Workshop agreed on five microsatellite markers for MSI testing, two mononucleotides and three dinucleotides: BAT25, BAT26, D2S123, D5S346 and D17S250 [21]. Tumors are defined as: MSI-High (two or more microsatellites are unstable), MSI-Low (one microsatellite out of five is unstable) and microsatellite stable [15, 21]. MSI testing has great clinical significance for cancer prevention, prognosis and treatment. For example, prognosis is good for many MSI-High colorectal cancer patients [22] and aspirin can prevent MSI in patients with germline mutations in *MSH2* and *MLH1* genes [23]. Treatment with 5-fluorouracil seems to be not effective in MSI-High patients [24], despite a report showing some benefit for stage IV MSI-High patients [25]. Immunotherapy has had promising results for MSI-High or MMR-deficient patients, which led the FDA to recently approve treatment regimens with the immunotherapeutic agent Keytruda (pembrolizumab), a PD-1 inhibitor [26].

## DNA MMR AND MUTATOR MICROORGANISMS

MMR gene mutations are observed in monocellular as well as multicellular organisms. In multicellular organisms, these mutations can cause cancer. In monocellular organisms, these mutations can offer an adaptive advantage through the ‘mutator’ phenomenon. Eucaryotic somatic cells with MMR gene mutations may have also increased fitness under the concept of ‘mutator’ cells. By virtue of the MMR mutation that may increase their fitness, mutator cells are also potentially cancerous cells.

The term ‘mutator’ is used for cells that have increased mutagenesis rate, which contributes to their survival under demanding or hostile environments. Most of the knowledge we have of this phenomenon comes from antibiotic-resistant bacteria or other drug-resistant microorganisms. Commonly, mutator microorganisms’ strains have a defective MMR pathway [27, 28]. *Escherichia coli* mutators were among the first that were studied [29]. In these cases, a partially defective MMR system is compatible with life and in fact may have beneficial effects for survival. If more mutations emerge after each cell division, then the probability increases for the appearance of a beneficial mutation which would allow the population to escape extinction. In evolutionary terms, antibiotics are catastrophic and highly selective for bacterial populations. If a mutator strain exists inside the population, this allows for tremendous adaptive capacities to increase replication after antibiotic treatment.

There are several examples of mutator strains. Studies show that MMR-deficient *Pseudomonas aeruginosa* is antibiotic-resistant and has increased virulence [30–32]. This has been a major problem for cystic fibrosis patients given that *P.aeruginosa* lung infections are a life-threatening condition for these patients. Generally, mutator multidrug-resistant bacterial strains are common in chronic infections, like cystic fibrosis or urinary tract infections. Patients in these cases receive multiple antibiotic cycles and bacteria are continuously under positive selection for antibiotic resistance [31, 33]. Antibiotic-resistant *Salmonella* strains have also been identified with mutations in MMR genes [29, 34]. Fungi is not an exception: MMR gene mutations have been found in *Cryptococcus*, *Candida* and *Aspergillus* genus, all of which are characterized by increased mutagenesis rates and rapid adaptation to antifungal drugs [28, 35].

## ANTIBIOTIC STRESS AND SOMATIC EVOLUTION

### Somatic evolution favors cancer mutations in healthy tissues

Recent advances in genomic analysis of somatic tissues challenge the standard knowledge that somatic mutations in oncogenes and tumor suppressor genes are always pathogenic. Mutations in oncogenes and tumor suppressor genes can lead to clonal expansions and adaptation in cells harboring these mutated genes. Martincorena *et al*. [36] found thousands of mutations in esophageal tissue from healthy individuals, including mutations in 14 well-known cancer genes. Most of the mutations were on the *NOTCH1* and *TP53* genes, the most frequently mutated ones in esophageal cancer. *NOTCH1* mutations in normal esophagus were several times higher than in esophageal cancers. Similar results were published soon after for many other healthy human tissues like endometrial, colorectal and liver [37–39]. Many ‘driver’ mutations in cancer genes in those healthy tissues were found to be under positive selection. It remains unknown why these individuals do not develop cancer. As such, it is obvious that the external environment drives selection and evolution even in normal somatic cells. The hypothesis offered in this perspective is that cells that are resistant to apoptosis or have a high mutagenesis rate have an evolutionary advantage under stressful conditions, such as those conferred by drugs (e.g. antibiotics), poisons, oxidative stress, starvation, cold, etc. This may be the way that our cells survive under diverse and challenging conditions.

### Antibiotics may induce somatic selection for mutator cells

Intestinal epithelial cells with mutator capabilities have an adaptive advantage under stressful conditions, e.g. anticancer therapy [40]. MMR-deficient intestinal cells probably experience positive selective pressures in such stressful environments. This is a procedure that mimics selection of MMR-deficient bacteria or other monocellular organisms under harsh conditions. As it was described in the previous sections, antibiotics cause an intense evolutionary pressure, benefiting mutator selection. Pharmacokinetic studies show that 20–60% of the orally administered tetracyclines’ dose is excreted through the feces [41]. As a result, oral treatment exposes gastrointestinal cells to a high antibiotic concentration for a prolonged time. This exposure can potentially cause stress to colon mucosa cells. Antibiotics have a high specificity for bacterial proteins or nucleic acids, but this is not absolute. The low-grade affinity of antibiotics with human biomolecules can cause significant cell toxicity, especially if given in multiple treatment cycles (or for a prolonged time). The following antibiotic categories are of high concern: antibiotics that bind the bacterial large 50S ribosomal unit (e.g. macrolides), antibiotics that bind the bacterial small 30S ribosomal unit (e.g. aminoglycosides), antibiotics that inhibit bacterial tRNA biogenesis or function (e.g. tetracyclines). The bacterial large and small ribosomal units are not completely unrelated with the human ones. Similarities exist and these antibiotics can potentially inhibit protein synthesis and become toxic for human cells [42, 43]. Enzymes that participate in bacterial tRNA biosynthesis or function also share some homology with the human versions. Again, tRNA antibiotics can potentially be toxic for humans, by inhibiting protein synthesis [44].

Under repeated antibiotic courses, MMR-deficient colon mucosa cells can be selected as in the case of bacteria. These evolutionary pressures probably affect colon crypt stem cells, which are small clonal units occupying intestinal spaces referred to as crypts. Mutations in non-stem cells usually do not accumulate since they have limited life spans, while the stem cells are responsible for cell proliferation of the crypt. Despite stem cells being quite resistant to mutagenesis, inevitably mutations appear during ageing [45]. Crypt stem-cells may increase their stress-tolerance with or without mutations but in the latter case, MMR pathway can be involved simply through downregulation of MMR gene expression. Russo *et al*. [46] showed that this is the case with colon cancer cells under anticancer therapy stress. EGFR inhibition induced a negative regulation of MMR gene expression. MMR mutations or expression inhibition is a possible explanation for the association of antibiotics use with colon cancer.

## TESTING THE HYPOTHESIS—CONCLUSION

**Figure 2.**
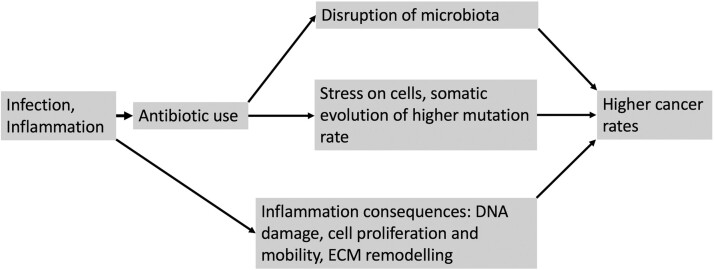
Possible ways that inflammation or antibiotic use can predispose to cancer. ECM, extracellular matrix.

Studies have reported a gut microbiome imbalance (dysbiosis) in patients with colorectal cancer, showing an increase of the population of ‘bad’ microbes compared to a decrease of ‘good’ microbes [47]. In light of these studies, the association of antibiotics with colon cancer has been attributed to microbiome imbalance. According to the perspective offered here, an evolutionary explanation must be considered as playing a significant role in the carcinogenic potential of antibiotics. However, other explanations may exist as well. Inflammation caused by infection is also a cause of cancer. Obviously, patients who needed treatment by antibiotics have suffered by an infection. It is known that inflammation is accompanied by immune cells infiltration, fibroblast recruitment and activation, and extracellular matrix remodeling. These procedures involve cell proliferation, increased cell mobility and increased cell penetrance. Subsequently, carcinogenesis probabilities are increasing, especially on the background of genetic variants on tumor suppressor genes or oncogenes [48]. Additionally, people who take antibiotics frequently may have less effective immune systems. Immune system is responsible for eliminating cancer cells in our body. People with weak immune systems, e.g. HIV infected people, are highly predisposed for cancer. Many other factors can also to be considered, like diet and other drugs taken together with antibiotics. A specific diet could be associated with cancer if some of its components are chemically interfering with antibiotics. Patients frequently taking antibiotics may also take frequently other kind of drugs. These can add on their risk for cancer. Despite the fact that most of the studies have considered parameters like smoking and alcohol, residual confounding may be present, e.g. units of alcohol consumed, number of packs per year for smokers, etc. [49]. Of course, it is extremely difficult for a study to adjust for all these parameters. Figure 2 summarizes the major factors discussed here that can drive to cancer.

The MMR genes’ hypothesis could be tested by multiple ways. A population-based study would be ideal, by arranging prospective cohorts of patients treated frequently with antibiotics. Steps: (i) Patients undergo once a year colonoscopy examination, checking for any alterations in their colon mucosa, (ii) biopsies must be taken from any abnormal forms of tissue, like polyps or cancer-like malformations, (iii) DNA from those tissues will be tested for MSI, (iv) exome sequencing can be performed in polyp DNA or tumor DNA, looking for mutations in MMR genes or other implicated genes, (v) groups of cancer patients with an already MSI-tested biopsy, can be examined for a previous history of multiple antibiotic treatments, comparing the MSI-positive and the MSI-negative ones.

The weakness of testing this hypothesis in humans is the need of colonoscopy. Colonoscopy is considered an invasive method, and this may be problematic under a research protocol. Additionally, biopsy testing cannot differentiate between direct and indirect effects of antibiotics. An alternative way to test this hypothesis is the use of animal models. Mice and zebrafish can be used as well. Steps: (i) Antibiotics can be administered in mice or zebrafish for a prolonged time, (ii) After some months (multiple time points can be used), DNA from multiple cell types, including intestinal cells, could be checked for any MMR gene pathogenic mutations, (iii) Results must be compared with antibiotic-free animals of the same age. Experiments can be designed to be more complicated, e.g. by performing comparisons between microbiota-free animals vs normal microbiota animals. Additionally, cancer incidence must be estimated, between treated and non-treated animals.

Similar experiments can be performed in cell cultures, preferably colon cell tissue cultures. Cultured cells treated for a prolonged time with antibiotics and antibiotic-free cells can be tested for MMR gene mutations. More advanced technology can be used like organ-on-a-chip models as well. Microfluidic organ-on-a-chip models of human intestine are available [50]. Chip experiments can be performed as described above, followed by MMR gene re-sequencing. Again, appropriate comparisons must be designed, e.g. intestine cell chips treated with antibiotics vs antibiotic-free chips, microbiota intestine cell chips vs microbiota-free intestine cell chips, etc.

The above suggestions can confirm or reject the hypothesis of MMR-deficient mutator cell selection. In addition, it would be important to consider whether extensive use of antibiotics by cancer patients could be risky as MSI-negative tumors can be transformed to MSI-positive after exposure to a harsh micro-environment. These tumors are more aggressive than the previous ones. It is probably wise for cancer patients to carefully consider antibiotic treatments or generally drugs that can increase death resistance of their cells.

In conclusion, an evolutionary explanation is proposed for the association of antibiotics with colorectal cancer, which has been revealed in multiple large-scale population-based studies. Testing this hypothesis is feasible, especially in national cancer reference centers, where large cohorts of patients exist. Somatic selection is the key for the understanding of many conditions related with human disease.
